# 2D Graphene Oxide Membrane Nanoreactors for Rapid Directional Flow Ring‐Opening Reactions with Dominant Same‐Configuration Products

**DOI:** 10.1002/advs.202308388

**Published:** 2024-02-28

**Authors:** Jiangwei Fu, Shuai Pang, Yuhui Zhang, Xiang Li, Bo Song, Daoling Peng, Xiqi Zhang, Lei Jiang

**Affiliations:** ^1^ CAS Key Laboratory of Bio‐Inspired Materials and Interfacial Science Technical Institute of Physics and Chemistry Chinese Academy of Sciences Beijing 100190 P. R. China; ^2^ School of Future Technology University of Chinese Academy of Sciences Beijing 100049 P. R. China; ^3^ University of Chinese Academy of Sciences Beijing 100049 P. R. China; ^4^ School of Optical‐Electrical Computer Engineering University of Shanghai for Science and Technology Shanghai 200093 P. R. China; ^5^ Science and Technology Center for Quantum Biology National Institute of Extremely‐Weak Magnetic Field Infrastructure Hangzhou 310051 P. R. China; ^6^ Key Laboratory of Theoretical Chemistry of Environment Ministry of Education School of Environment South China Normal University Guangzhou 510006 P. R. China; ^7^ Binzhou Institute of Technology Weiqiao‐UCAS Science and Technology Park Binzhou 256600 P. R. China

**Keywords:** chiral conversion, directional flow, graphene oxide membranes, nanoconfinement, ring‐opening reaction

## Abstract

Nanoconfinement within enzymes can increase reaction rate and improve selectivity under mild conditions. However, it remains a great challenge to achieve chemical reactions imitating enzymes with directional molecular motion, short reaction time, ≈100% conversion, and chiral conversion in artificial nanoconfined systems. Here, directional flow ring‐opening reactions of styrene oxide and alcohols are demonstrated with ≈100% conversion in <120 s at 22 °C using graphene oxide membrane nanoreactors. Dominant products have the same configuration as chiral styrene oxide in confined reactions, which is dramatically opposed to bulk reactions. The unique chiral conversion mechanism is caused by spatial confinement, limiting the inversion of benzylic chiral carbon. Moreover, the enantiomeric excess of same‐configuration products increased with higher alkyl charge in confined reactions. This work provides a new route to achieve rapid flow ring‐opening reactions with specific chiral conversion within 2D nanoconfined channels, and insights into the impact of nanoconfinement on ring‐opening reaction mechanisms.

## Graphical abstract

This work reports directional flow ring‐opening reaction occurring in 2D nanochannels of graphene oxide membranes. Compared with the bulk reaction, nanoconfinement alters the path and lowers the reaction activation energy barrier, thereby achieving ≈100% conversion at 22 °C with a short reaction time (<120 s) and favoring the generation of products with same‐configuration.

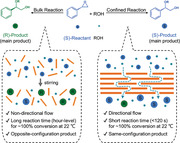

## 1 Introduction

Enzymes, catalysts created by nature, have confined active sites.^[^ ^1^ ^]^ Confinement within enzymes allows them to accelerate chemical reactions with remarkable efficiency,^[^ ^2^ ^]^ improve selectivity,^[^ ^3^ ^]^ and synthesize complex molecules under mild conditions.^[^ ^4^ ^]^ In order to imitate the behavior of enzymes in nature and explore the unique reactivity of molecules under confinement, a series of artificial nanoreactors such as coordination cage,^[^ ^5^, ^6^ ^]^ cucurbit,^[^ ^7^, ^8^ ^]^ cavitands,^[^ ^9^, ^10^ ^]^ carbon nanotubes,^[^ ^11^, ^12^ ^]^ zeolites,^[^ ^13^, ^14^, ^15^, ^16^, ^17^, ^18^, ^19^ ^]^ metal‐organic cage,^[^ ^20^ ^]^ covalent organic framework (COF),^[^ ^21^, ^22^, ^23^, ^24^, ^25^ ^]^ and metal‐organic framework (MOF)^[^ ^26^, ^27^, ^28^, ^29^, ^30^, ^31^, ^32^ ^]^ have been developed. It has been proved experimentally and theoretically that confinement effects in the nanoreactors can affect the kinetics, thermodynamics, and underlying mechanism of chemical reactions.^[^ ^33^, ^34^, ^35^ ^]^ However, in comparison with enzyme biosynthesis, research on confined catalysis is far from achieving chemical reactions with low energy consumption, high conversion, high specificity, and directional molecular motion.^[^ ^36^, ^37^, ^38^, ^39^ ^]^

2D materials have emerged during the last decade as the appropriate candidate to mimic enzymes due to their easy fabrication and excellent up‐scalability.^[^ ^40^, ^41^, ^42^, ^43^, ^44^, ^45^, ^46^, ^47^ ^]^ Among the plethora of available 2D materials, graphene oxide (GO) is extensively studied owing to its versatile oxygen‐containing functional groups and ease of dispersibility in various solvents.^[^ ^48^, ^49^ ^]^ GO nanosheets can be assembled into membranes with laminar structure and the nanochannels of GO membranes display unique, precise, and ultra‐fast molecular transport properties.^[^ ^50^, ^51^, ^52^, ^53^, ^54^, ^55^ ^]^ Although the interlayer nanochannel theoretically is suitable to be confined space, constructing GO membrane nanoreactors for confined reactions is still rarely investigated.

Ring‐opening of epoxides with alcohols under acidic conditions is an important route to synthesize β‐alkoxy alcohols, which are precursors for a broad range of pharmaceuticals.^[^ ^56^, ^57^, ^58^ ^]^ Over the past decades, various artificial nanoreactors have been applied to ring‐opening of epoxides such as metal complexes, COF, and mesoporous silica.^[^ ^56^, ^59^, ^60^ ^]^ However, the application of these catalysts are usually limited by complicated preparation processes, high reaction temperature, long reaction time, and etc. The surface of GO comprises abundant oxygen‐containing functional groups such as epoxy, hydroxyl, and carboxyl groups,^[^ ^61^, ^62^ ^]^ which confer it certain acidity, suggesting the feasibility of catalyzing ring‐opening reactions. Moreover, chemical reactions occurring in the nanochannels of GO membranes are influenced by confinement effects, potentially leading to more efficient and special selective flow reactions in extremely short time.

Herein, GO membranes were prepared by vacuum filtration of GO nanosheets dispersion, and used as nanoreactors for ring‐opening flow reactions of styrene oxide with different alcohols. Driven by pressure difference, styrene oxide molecules move directionally through the GO nanochannels and react with alcohol molecules that also act as solvents. The ring‐opening reaction of styrene oxide with various alcohols is realized with ≈100% conversion in less than 120 s at 22 °C. Subsequently, we assessed the nanoconfined effect on the stereoselectivity of the ring‐opening reaction. The results showed that the dominant products within GO membrane nanoreactors had the same benzylic chiral carbon configuration as chiral styrene oxide, while dominant products with significantly opposite stereoscopic configuration were obtained through bulk reactions using GO nanosheets. Furthermore, the mechanisms governing the stereoselectivity of chemical transformations within GO membrane nanoreactors were revealed and the confinement effect on the overall reaction energy level change was investigated by density functional theory (DFT).

## 2 Results and Discussion

**Figure 1:**
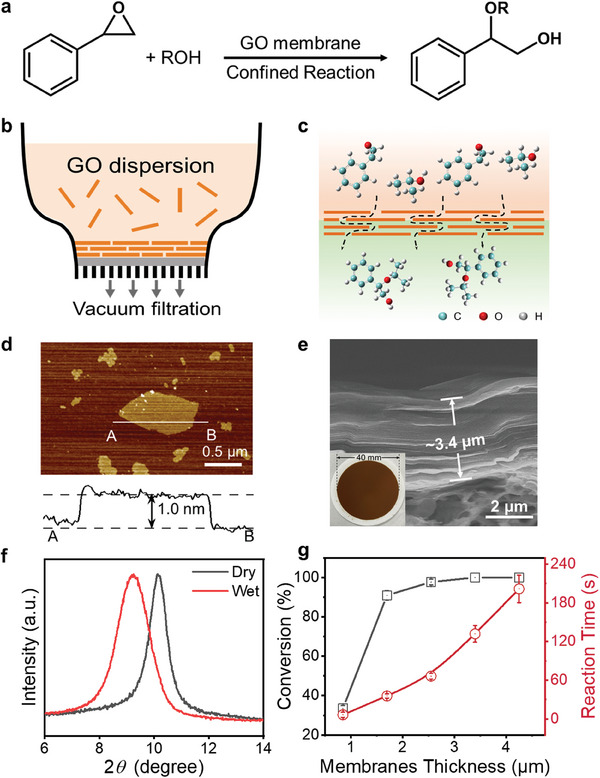
Fabrication and characterization of graphene oxide (GO) membrane and its catalytic performance for confined ring‐opening reaction of styrene oxide with alcohol. a) Scheme of the ring‐opening reaction between styrene oxide and alcohol catalyzed by GO membrane. b) Scheme of vacuum filtration process for the fabrication of GO membranes. c) Schematic diagram of GO membrane nanoreactors, showing the directional flow ring‐opening reaction of styrene oxide and isopropanol at 22 °C. d) AFM image and height profile of the GO nanosheets. e) The photograph of the as‐prepared GO membrane and its SEM image of the cross‐sectional view. f) XRD spectra of GO membranes (dry state and wet state). Due to the effect of isopropanol swelling, the d‐spacing of the GO membrane increases in the wet state. g) Conversion and reaction time of ring‐opening reaction between styrene oxide and isopropanol as a function of GO membranes thickness.

As shown in **Figure** **1**a, GO membranes were used to catalyze the ring‐opening reaction of styrene oxide with alcohols as both the solvent and nucleophile. The existence of oxygen‐containing groups on the surface of GO was confirmed by Fourier transform infrared (FTIR) spectra and X‐ray photoelectron spectroscopy (XPS) (Figures S1 and S2, Supporting Information). The absorption peaks at 1054 and 1731 cm^−1^ assigned to the C─O stretching of the alkoxy and C═O stretching modes of the carboxylic groups (acid sites) respectively, indicating the potential for catalyzing ring‐opening reactions.^[^ ^63^ ^]^ GO membranes were fabricated through the stacking of GO nanosheets onto nylon substrates by vacuum assisted filtration (Figure 1b) and employed as nanoreactors for catalyzing ring‐opening reaction (Figure 1c). Atomic force microscopy (AFM) characterization demonstrates that the thicknesses of GO nanosheets are ≈1.0 nm, which is consistent with a previous report,^[^ ^64^ ^]^ indicating that GO nanosheets are single‐layer structures (Figure 1d). Scanning electron microscopy (SEM) characterization on the cross‐section of the GO membrane also reveals the lamellar structure with a thickness of about 3.4 µm (Figure 1e). X‐ray diffraction (XRD) results indicate that dry‐state GO membranes have characteristic peaks at 2θ = 10.15°, which corresponds to the interlayer spacing (d‐spacing) of 8.70 Å. Due to the swelling effect of solvent,^[^ ^65^ ^]^ the in situ wet‐state d‐spacing of GO membranes increased 0.87 Å compared to the dry‐state d‐spacing (Figure 1f). Other alcohol solvents (ethanol, propanol, isobutyl alcohol, and etc.) have similar swelling effects on GO membranes (Figure S3 and Table S1, Supporting Information).

First, the catalytic activity of GO membrane nanoreactors toward the ring opening of styrene oxide with isopropanol was studied. Driven by the pressure difference, isopropanol acts as both reactants and solvents, reaction solution containing styrene oxide (0.25 m) permeates directionally through the GO membranes at 22 °C. Styrene oxide molecules react with surrounding isopropanol molecules in the interlayer nanochannels and the products flow out with the isopropanol solvent and return to the bulk phase. The flow volume of the reaction solution is linear as a function of time, indicating that the reaction solution flows steadily (Figure S4, Supporting Information). The conversion was confirmed by analysis of the collected solution through proton nuclear magnetic resonance (^1^H NMR) analysis. The results showed that the characteristic peaks of styrene oxide disappeared completely and the characteristic peaks of the products appeared, indicating that the reactants were completely transformed into products, and a ≈100% conversion of styrene oxide was achieved (Figure S5, Supporting Information).

In order to assess the effect of membrane thickness on the conversion and reaction time, GO membranes with different thicknesses were prepared. Five types of GO membranes with multilayer structures were fabricated by adjusting the filtration volume of the GO nanosheets dispersion. The membranes have multilayer structures with different thicknesses (Figure 1e and Figure S6, Supporting Information). The cross‐sectional thicknesses were 0.9, 1.7, 2.6, 3.4, and 4.2 µm, respectively. As shown in Figure 1g, the conversion of styrene oxide improves with the thickness of membranes increases, and the reaction time prolongs correspondingly. It can be seen that the conversion is poor when the membrane thickness is only 0.9 µm. This may be due to the short retention time of the reactants in the nanochannel, which leads to less possibility of reactants reacting with each other. When the membrane thickness is 3.4 µm, a ≈100% conversion of styrene oxide was achieved in a very short reaction time of less than 120 s.^[^ ^66^ ^]^ This can be explained that the overall contact time between the reactants and the active sites of the GO membrane nanoreactors increases with the increase of membrane thickness.

As a comparison, the bulk reaction of styrene oxide and isopropanol was carried out with dispersed GO nanosheets as heterogeneous catalysts under the same magnitude and conditions as the confined reaction. The bulk reaction is limited by the stirring time due to the high degree of freedom of the reactant molecules and the disordered diffusion in solution. It took ≈4.5 h for the bulk reaction to reach a ≈100% conversion of styrene oxide at 22 °C (Figure S7, Supporting Information), while the time for the confined reaction is less than 120 s. Moreover, the confined ring‐opening reactions of styrene oxide with ethanol, propanol, and isobutyl alcohol were tested by further extending the substrate scope. All confined reactions were able to achieve ≈100% conversion with reaction time ranging from <17 s to <43 s, which is significantly shorter than the time required for bulk reactions. (**Table** **1** and Figures S8–S11, Supporting Information).

**Table 1:** Comparison of bulk reaction and confined reaction.

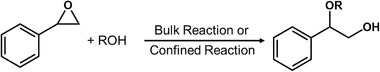			
Reaction condition^a)^	ROH	Conversion [%]^b)^	Time [s]
Bulk Reaction	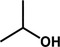	≈100^c)^	≈16200
Confined Reaction	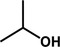	≈100^d)^	<120^e)^
Bulk Reaction	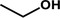	≈100^c)^	≈4200
Confined Reaction	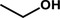	≈100^d)^	<43^e)^
Bulk Reaction	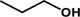	≈100^c)^	≈3000
Confined Reaction	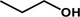	≈100^f)^	<34^e)^
Bulk Reaction	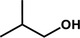	≈100^c)^	≈4200
Confined Reaction	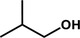	≈100 ^g)^	<17^e)^

^a)^ Reaction conditions: styrene oxide (0.25 m) in alcohol solvent at 22 °C;

^b)^ The conversion of styrene oxide was confirmed by analyzing the ^1^H NMR spectroscopy of collected solution;

^c)^ 1.5 mg GO nanosheets was used, which was in excess relative to the confined reaction;

^d)^ GO membrane with thickness of 3.4 µm was used;

^e)^ The time was calculated by the formula: $t_{R}=\frac{V_{M}}{k}=\frac{A\times h}{k}$, where V_M_ is the total volume of the membranes; k is slope of the reactant flow volume as a function of time; A is the channel cross‐sectional area of microfiltration devices ($A=\frac{\pi }{4}\times 15^{2}=176.7\;{\mathrm{mm}}^{2}$); h is the thickness of membrane which was confirmed by SEM characterization;

^f)^ GO membrane with thickness of 2.6 µm was used;

^g)^ GO membrane with thickness of 1.7 µm was used.

**Figure 2:**
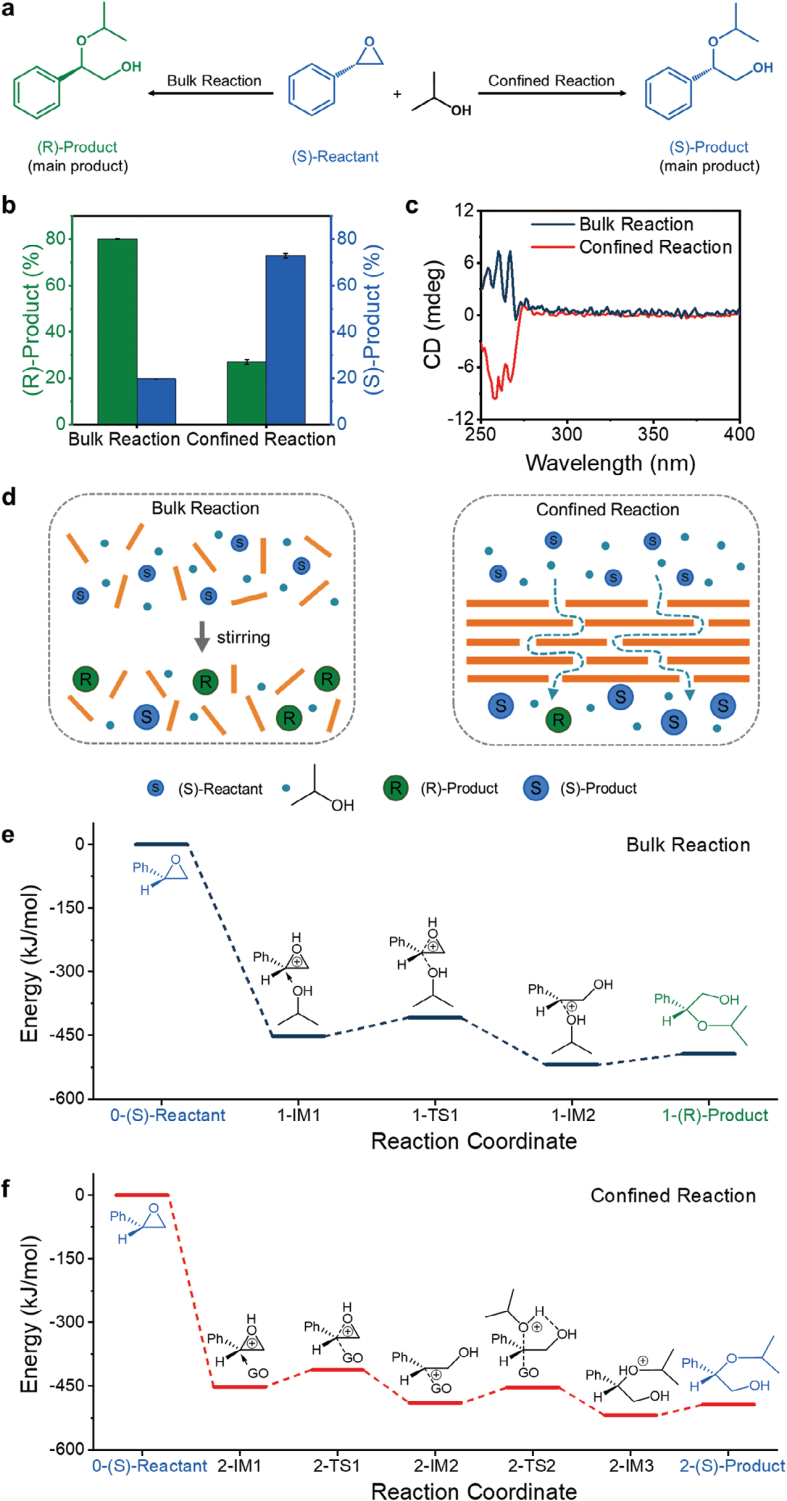
Reaction stereoselectivity comparison and density functional calculations (DFT) of the ring‐opening reaction of (S)‐styrene oxide with isopropanol under bulk and confined conditions. a) Schematic diagram showing the ring‐opening reaction of (S)‐styrene oxide with isopropanol under bulk and confined conditions obtaining main products ((R)‐product and (S)‐product) with different stereoconfiguration. b) Percentage of (R)‐product and (S)‐product obtained under bulk and confined reactions. c) CD spectra of the product obtained from the bulk reaction and confined reaction, respectively. d) Schematic illustration showing the difference in the stereoselectivity of (S)‐styrene oxide under bulk and confined reactions. e,f) Reaction paths of bulk reaction (e) and confined reaction (f) calculated by DFT. The labels TS and IM indicate the transition state and intermediate of the reaction, respectively.

Besides improving reaction efficiency, nanofinement may enhance or alter the selectivity for certain products over others. To investigate the nanoconfined effect within GO membrane nanoreactors on the stereoselectivity, the ring‐opening reaction of (S)‐styrene oxide with isopropanol was performed. Due to the inversion of the chiral carbon configuration or not, two products, the (R)‐product and the (S)‐product, were produced (**Figure** **2**a). The high‐performance liquid chromatography (HPLC) results showed that the ring‐opening reaction of (S)‐styrene oxide with isopropanol under confined conditions produced 27.1% (R)‐products and 72.9% (S)‐products. In contrast, the bulk reaction yielded 80.2% of (R)‐products and 19.8% of (S)‐products (Figure 2b and Figure S12, Supporting Information).

The difference in stereoselectivity was further confirmed by circular dichroism (CD), where the products obtained from the bulk and confined reactions had the same absorption peak positions and opposite signals (Figure 2c). It can be concluded that the stereogenic center was maintained under confined conditions, resulting in the dominant formation of the (S)‐product with the same configuration. In contrast, the (R)‐product with opposite configuration was the dominant product under bulk conditions due to the inversion of the stereogenic center (Figure 2d). Furthermore, the ring‐opening reaction of (R)‐styrene oxide with isopropanol was tested (Figure S13, Supporting Information), and the results showed that (R)‐product (same configuration) dominated under confined conditions where the stereogenic center is maintained, while the opposite configuration was prepared for the bulk reactions (Figure S14, Supporting Information). It can be inferred that the nanoconfined effect is the main factor affecting the stereoselectivity of the reaction, rather than the selective transport of products of a certain configuration by the membrane reactors.

These differences in reactivity as well as stereoselectivity can be attributed to the nanoconfined effect within GO membrane reactors, which affect the reaction paths and mechanisms (Figure 2e,f). The ring‐opening reaction of (S)‐styrene oxide with isopropanol can be summarized as three steps which are protonation, nucleophilic reagent attack and deprotonation. For both bulk and confined reaction, the acidity possessed by GO nanosheets causes the oxygen atom of (S)‐styrene oxide to be protonated and positively charged via solvent‐assisted proton conduction. The energies from the reactant (0‐(S)‐Reactant) to the first intermediate (IM1) for the bulk and confined conditions are calculated to be −451.7 kJ mol^−1^, indicating the protonation process is barrier‐free. The process of protonation causes the oxygen atom to attract electrons to the adjacent cyclic carbon atom, resulting in a weakened C─O bond and a partially positively charged cyclic carbon atom, thereby increasing the ability to bind to nucleophilic reagents. Subsequently, the nucleophilic reagent attacks the ring carbon atom with more substituents, as it helps to disperse the positive charge to stabilize the system. For the bulk reaction, the isopropanol acts as a nucleophilic reagent and the lone pair of electrons of its oxygen atom attacks the ring carbon atom from the back side to produce the second intermediate (1‐IM2). A transition state (1‐TS1) is thus required from 1‐IM1 to 1‐IM2 and the Gibbs free energies of 1‐TS1 and 1‐IM1 are −408.2 and −451.7 kJ mol^−1^, resulting in an energy barrier of 43.5 kJ mol^−1^. For the confined reaction, the hydroxyl content on the surface of the GO nanosheets is greater than the content of isopropanol molecules in the 2D channel of the GO membrane nanoreactors. Therefore, the hydroxyl group on the GO surface acts as a nucleophilic reagent, attacking the ring carbon atom from the back side to obtain the second intermediate (2‐IM2). The energy difference of the first transition state (2‐TS1) and the first intermediate (2‐IM1) is 39.2 kJ mol^−1^, indicating that the energy barrier is lower than that of the bulk reaction. Therefore, the (S)‐styrene oxide is more favorable to interact with the hydroxyl group on the GO surface due to the lower energy barrier from IM1 to TS1 of confined reaction. And then, due to the spatial site resistance of GO, the lone pair of electrons of alcohol‐oxygen attack the cyclic carbon atom from the front side to generate the third intermediate (2‐IM3). A second transition state (2‐TS2) is required from 2‐IM2 to 2‐IM3 and the energies of 2‐TS2 and 2‐IM2 are −453.2 and −489.1 kJ mol^−1^, resulting in an energy barrier of 35.9 kJ mol^−1^.

Subsequently, the final product is obtained through a deprotonation process with the alcohol solvent as proton acceptor, resulting in an energy barrier of 25.3 kJ mol^−1^. The S_N_2 reactions result in a chiral change and the confined reaction pathway undergoes S_N_2 reactions twice, therefore the bulk reaction route producing the (R)‐product and the confined reaction pathways generating the (S)‐product. The above analysis shows that the formation of the first transition state led to a maximum energy barrier that primarily determined the reaction rate. And the lower activation energy barrier of the confined reaction achieves a higher‐efficiency reaction performance and more products of same‐configuration maintained.

To verify the effect of hydroxyl groups on the reaction stereoselectivity of GO surface, the GO membranes with the thickness of 3.4 µm were thermally treated in an oven at 120 °C for 12 h, named GO‐120 °C. The chemical composition of GO‐120 °C membranes was analyzed by XPS (Figure S15, Supporting Information). Figure S16 and Table S2, Supporting Information show the relative ratio of various carbon atoms in the high‐resolution C 1s spectra that the C─O intensity decreases after treatment at 120 °C relative to GO membranes, indicating the breaking of partial C─O bonds. Compared to GO membranes, FTIR spectra of the GO‐120 °C membranes shows a weaker peak at ≈3408 cm^−1^ that attributed to the stretching vibration mode of hydroxyl group (Figure S17, Supporting Information). GO‐120 °C membranes were used to catalyze the ring‐opening reaction of (S)‐styrene oxide with isopropanol and exhibited a lower stereoselectivity (Figure S18, Supporting Information). The membrane thickness and wet‐state d‐spacing of GO‐120 °C membranes are comparable to that of the GO membrane (Figure S19, Supporting Information). Therefore, the lower stereoselectivity obtained by GO‐120 °C membranes may be attributed to a decrease in hydroxyl content that results in an increase opportunity for isopropanol to react with chiral styrene oxide in the 2D confined channel, which is not favorable for the reaction to proceed toward the confined route. To explore the effect of membrane thickness on stereoselectivity of the reaction, GO membranes with the thickness of 1.7 µm and corresponding GO‐120 °C membranes were prepared and used as membrane reactors to catalyze the ring‐opening reaction of (S)‐styrene oxide with isopropanol. Compared to GO membranes, GO‐120 °C membranes exhibited a lower enantiomeric excess which is consistent with the results obtained by membranes of 3.4 µm (Figure S20, Supporting Information). Therefore, the influence of membrane thickness on enantiomeric excess is insignificant.

**Figure 3:**
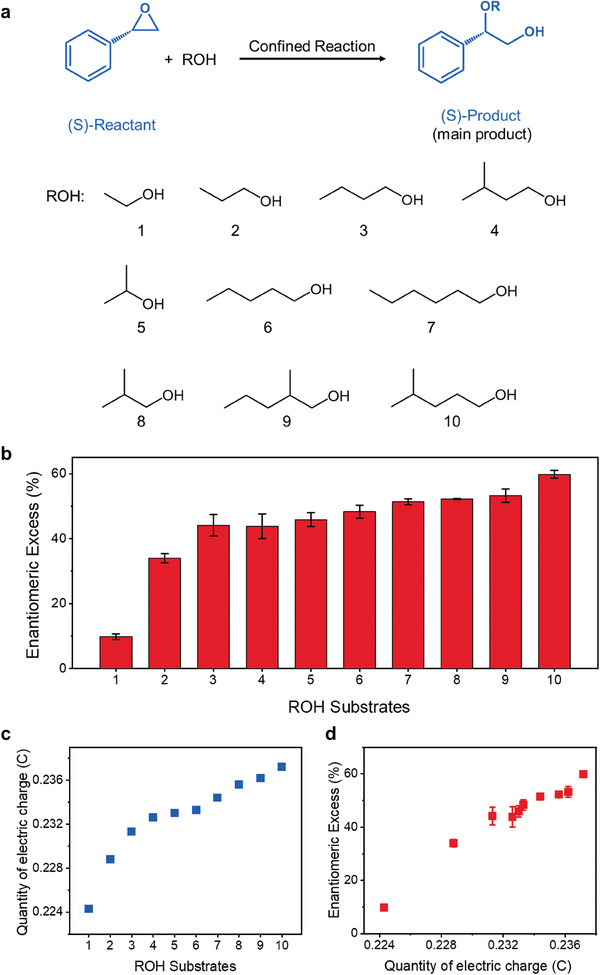
Alkyl alcohol substrates extension and the effect of electric charges of alkyl substituents on stereoselectivity in the confined reaction. a) Schematic diagram of the confined reaction of (S)‐styrene oxide with different alkyl alcohols. b) Enantiomeric excess of ring‐opening reaction between (S)‐styrene oxide and different alkyl alcohol substrates. c) DFT calculations of electric charge of different alkyl (R) groups. d) Enantiomeric excess of ring‐opening reaction between (S)‐styrene oxide and alkyl alcohols under confined conditions as a function of electric charges of alkyl substituents.

For the confined reaction, the overall five‐membered ring formed in the 2‐TS2 was positively charged. Therefore, increasing the electric charge of alkyl (R) groups can reduce the energy of 2‐TS2 and stabilize its structure. The lower the energy barrier from 2‐IM2 to the 2‐TS2, the faster the reaction rate and the more the corresponding products (2‐(S)‐Product). The stereoselectivity of the ring‐opening reactions of (S)‐styrene oxide with different alkyl alcohols was tested (**Figure** **3**a and Figures S21–S29, Supporting Information). These solvents also have similar expansion effect on GO membranes, and the influence of d‐spacing variation on confined reactions can be ignored (Figure S3 and Table S1, Supporting Information). Figure 3b showed that enantiomeric excess of ring‐opening reaction between (S)‐styrene oxide and different alkyl alcohol substrates. The total electric charge of different alkyl groups was calculated by DFT as shown in Figure 3c. The results showed that the stereoselectivity of the reaction increased with the increase of the electric charge carried by the alkyl substituents, and the enantiomeric excess was nearly linearly related to the quantity of electric charge (Figure 3d). In addition, the surface of GO contains abundant oxygen‐containing functional groups, resulting in a negatively charged environment on its surface according to the zeta potential measurement (Figure S30, Supporting Information). This environment can stabilize 2‐TS1 and 2‐TS2 structures with positive electric charge and reduce reaction energy. Due to the surface charge interactions, the increase in the electric charge of near‐linear alkyl chains enhances their horizontal movement in a 2D confined nanochannel,^[^ ^67^, ^68^ ^]^ which spatially promotes the formation of 2‐TS2 and same configuration products. The consistent wet‐state d‐spacing of GO membranes in different alcohol conditions also supports this point.

As for the bulk reaction, the increase in the electric charge of the alkyl oxygen (RO) group leads to a decrease in the enantiomeric excess value of the dominant opposite‐configuration (R)‐Product (Figures S31 and S32, Supporting Information). The increased electric charge of alkyl oxygen groups and larger substituents may impact S_N_2 reactions, due to the absence of spatial confinement and the dual role of alcohol molecules as reactants and solvents. The S_N_2 reactions remain dominant, but the increasing proportion of S_N_1 reactions decreases the enantiomeric excess value of the (R)‐product in bulk reactions. Therefore, we can conclude that the influence of substituents on enantiomeric excess is different between the confined reaction and bulk reaction. The enantiomeric excess in the confined reaction increases with the increase of electric charges of larger alkyl substituents, while that in the bulk reaction decreases with the increase of electric charges of larger alkyl oxygen substituents.

## 3 Conclusion

In this work, we fabricated GO membrane nanoreactors by vacuum filtration and applied them to catalyze the flow ring‐opening reaction of styrene oxide with alcohols at 22 °C. Compared with the bulk reaction, nanoconfinement effect improves the reaction rates, shortens the reaction times, and exhibits excellent catalytic properties for many substrates. Simultaneously, ring‐opening reactions under confined conditions exhibit different stereoselectivity from that of the bulk conditions. Under confined conditions, same configuration of chiral carbon is more inclined to be maintained, whereas is opposite configuration under bulk conditions. Theoretical results demonstrated the confined effect within GO membrane reactors alters the path and mechanism of the ring‐opening reaction and lowers the reaction activation energy barrier, thereby favoring the generation of products with same configuration. The higher the electric charge of the alkyl substituents in alcohols, the greater the enantiomeric excess of confined reactions. Utilizing 2D interlayer confinement, oriented molecule flow in continuous laminar membranes offers the possibility of achieving flow ring‐opening reactions with high reaction rate and chiral conversion.

## 4 Experimental Section

### Materials

Single‐layer graphene oxide dispersion (GO, >500 nm, 2 mg mL^−1^, solvent: water) was purchased from XFNANO (Nanjing, China). Styrene oxide, S‐styrene oxide, R‐styrene oxide, propanol, isopropanol, 1‐butanol, isobutanol, 1‐pentanol, 1‐hexanol, 3‐methyl‐1‐butanol, 4‐methyl‐1‐pentanol, 2‐methyl‐1‐pentanol were purchased from TCI. Methanol (GR), ethanol (GR) and acetonitrile (GR) were purchased from Macklin. Chloroform‐d (99.8 atom%D, contains 0.03% v/v TMS) was purchased from J&K Scientific. These chemical reagents were used as received. Deionized water (18.2 MΩ cm, MilliQ) was used in the experiments.

### Characterizations

Fourier Transform Infrared (FTIR) spectra was recorded on a Thermo Scientific Nicolet IS20 FTIR Spectrometer, using KBr as a reference. X‐ray photoelectron spectroscopy (XPS) experiments was performed in an ultrahigh‐vacuum system (Thermo escalab 250Xi), and high‐resolution spectra were obtained by curve fitting of the synthetic peak components using the Avantage software. The surface topography images were obtained by an atomic force microscope (AFM, Bruker Dension Icon). The microstructure of GO membranes was characterized using a field‐emission scanning electron microscope (HITACHI S‐4800). The X‐ray diffraction (XRD) of samples were recorded using a Bruker D8 focus diffractometer using Cu Kα radiation (*λ* = 1.5406 Å). ^1^H NMR spectroscopy was recorded on a Bruker Advance 600 MHz NMR spectrometer using chloroform‐d as the solvent. High performance liquid chromatography (HPLC) was accomplished on a ThermoFisher vanquish HPLC System, using a Diacel Corporation Inc. Chiralpak IF‐3 column (3 µm particle size, 4.6 mm I.D. × 150 mm). Circular dichroism spectroscopy (CD) characterization was performed using a CD spectrometer (J‐815, JASCO, Japan).

### Preparation of GO Membranes

GO membranes with different thicknesses of 0.9, 1.7, 2.6, 3.4, and 4.2 µm were fabricated by adjusting the filtration volume of 1.5, 3, 4.5, 6, and 7.5 mL of GO nanosheets dispersions (2 mg mL^−1^), respectively. The certain volume of GO dispersion (2 mg mL^−1^) was diluted to 40 mL and ultrasonicated for homogeneous dispersion. The dispersion was vacuum filtered on the nylon filter film (47 mm in diameter, pore size of 0.22 µm) into a highly uniform membrane (40 mm in diameter). The as‐prepared membrane was air‐dried at room temperature for 12 hours and then used in the following reaction experiment.

### GO Membranes for the Confined Reaction

The surface of the GO membranes was purged with nitrogen to remove the floating dust. The flat and smooth part of the GO membranes was selected and cut to a square octagon. The GO membrane was fixed to the porous glass substrate surface of the microfiltration device, covered above with a glass measuring cylinder (inner diameter 15 mm) and clamped to ensure a tight seal. The effective mass of the catalyst is less one‐quarter of the mass of the prepared GO membrane. The reaction solution comprising the styrene oxide (0.25 m) and the alcohols (act as reactant and solvent) was then added to the upper measuring cylinder, the surface of which was covered with aluminum foil. Driven by the pressure difference (0.9 atm), the reaction solution flowed through the interlayer 2D nanochannels within the GO membranes, where the reactants reacted in the interlayer at 22 °C and the products exited the system with the solvent. The solvent is removed from the permeate by rotary evaporation and then dissolved with chloroform‐d. The composition was determined by ^1^H NMR and conversion was calculated. Accordingly, dissolving the collection fluid with the solvent mixture of acetonitrile and water, the composition was determined by HPLC and the enantiomeric excess was calculated.

### Measurement of Layer Spacing in the Wet State of GO Membranes

Due to the swelling effect of solvents, the wet‐state d‐spacing of GO membranes may be significantly larger than the d‐spacing under dry conditions. The preparation process of wet‐state GO membranes is similar to the reaction process, with the difference that the reaction solution is replaced with an alcoholic solvent. After a few hours, the wet‐state GO membrane was removed and immediately followed by XRD characterization.

### Calculation of Reaction Time of the Confined Reaction

Pang et al. calculated the retention time of solution through GO‐NH_2_ laminar membranes,^[^ ^35^ ^]^ we refer to their calculations with the following formula:

(1) $$t_{R}=\frac{V_{M}}{k}=\frac{A\times h}{k}$$

where V_M_ is the total volume of the membranes; k is slope of the reactant flow volume as a function of time; A is the channel cross‐sectional area of microfiltration devices, $A\;=\frac{\pi }{4}\;\times \;15^{2}=\;176.7\;mm^{2}$; h is the thickness of membrane. The meaning of t_R_ represents the time required for the reaction solution to fill the total volume of the GO membrane. Considering the thickness of monolayer graphene (≈0.34 nm), except the laminar nanochannels, the GO nanosheets occupy a portion of the total volume of the GO membrane. Therefore, the effective volume filled by the reaction solution is less than V_M_, which makes the actual reaction time less than t_R_. t_R_ is approximated to represent the reaction time of the interlayer confined reaction.

### Calculation of Enantiomeric Excess Value

The enantiomeric excess (ee) value of reaction was calculated from the peak areas of the product of each configuration, namely A_R_ ((R)‐product) and A_S_ ((S)‐product) using the equation below:

(2) $$\mathrm{ee}\;value\;\left(\%\right)=\frac{\left|A_{R}-A_{S}\right|}{A_{R}+\;A_{S}}\times 100\%$$

### Dispersed GO Nanosheets for the Bulk Reaction

The bulk reaction was carried out in a 10 mL glass flask. First, 1.5 mg GO nanosheets were dissolved in 3 mL of alcohol solution containing styrene oxide (0.25 m), which the catalyst is in excess relative to the membrane reaction. Then, the reaction was carried out at 22 °C under stirring conditions and samples were taken at intervals. The GO powder was removed by a needle filter and after rotary evaporation to remove the remaining solvent, the reaction conversion and stereoselectivity were determined by ^1^H NMR and HPLC.

### Methods of DFT Calculations

The density functional theory (DFT) calculations were performed on the Gaussian16 program. The M06‐2X density (exchange‐correlation) functional were employed as calculation method. The 6–31G(d) and 6–311+G(d,p) basis set were used in geometry optimization and single‐point free energy calculations, respectively. The SMD implicit model was used as solvation model. As a result, the value of the free energy change relative to the reactant was calculated.

### Statistical Analysis

The XRD and XPS data are pre‐processed with normalization.

## Conflict of Interest

The authors declare no conflict of interest.

## Supporting information

Supporting Information

## Data Availability

The data that support the findings of this study are available in the supplementary material of this article.
