# Supplementary material for: 2D Graphene Oxide Membrane Nanoreactors for Rapid Directional Flow Ring‐Opening Reactions with Dominant Same‐Configuration Products
Source: Adv Sci (Weinh). 2024 Feb 28;11(16):2308388. doi: 10.1002/advs.202308388 (PMC11040342; doi:10.1002/advs.202308388)
Supplement: Supplementary file 1 — Supporting Information [file ADVS-11-2308388-s001.pdf]

ADVANCED SCIENCE
Open Access

Supporting Information

for *Adv. Sci.*, DOI 10.1002/advs.202308388

2D Graphene Oxide Membrane Nanoreactors for Rapid Directional Flow Ring-Opening Reactions with Dominant Same-Configuration Products

Jiangwei Fu, Shuai Pang, Yuhui Zhang, Xiang Li, Bo Song, Daoling Peng, Xiqi Zhang* and Lei Jiang*

Supporting Information

2D Graphene Oxide Membrane Nanoreactors for Rapid Directional Flow Ring-opening Reactions with Dominant Same-configuration Products

Jiangwei Fu, Shuai Pang, Yuhui Zhang, Xiang Li, Bo Song, Daoling Peng, Xiqi Zhang,* and Lei Jiang*

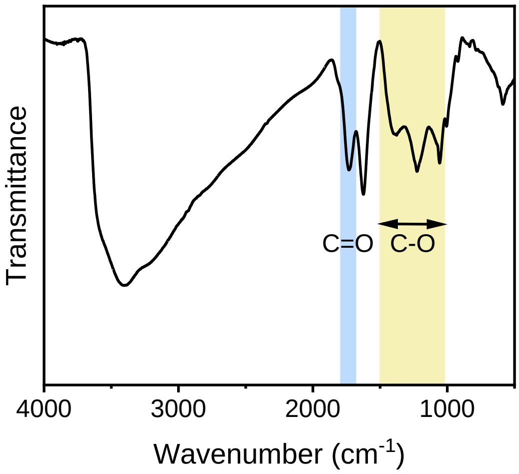

Figure S1. FTIR spectra of the GO. The absorption bands at 1384 cm^{-1} and 1225 cm^{-1} show the C-O stretching of the carboxy and epoxy, respectively.[1, 2]

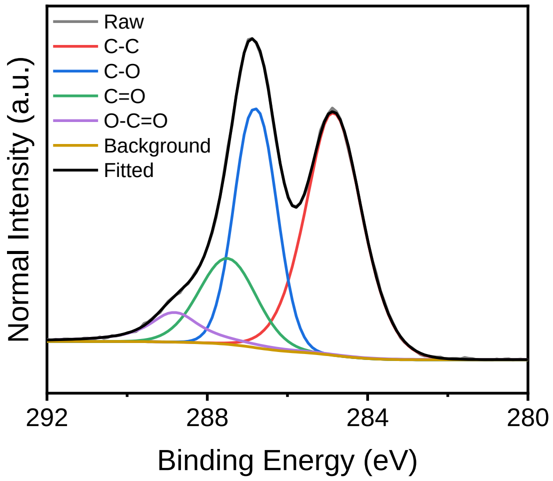

Figure S2. C 1s XPS spectra of the GO, indicating the existence of surface oxygen-containing functional groups. The peaks at approximately 284.8, 286.8, 287.4 and 288.7 eV were attributed to the C–C, C–O, C=O (carbonyl C) and O=C–OH (carboxyl C) functional groups, respectively.[3-6]

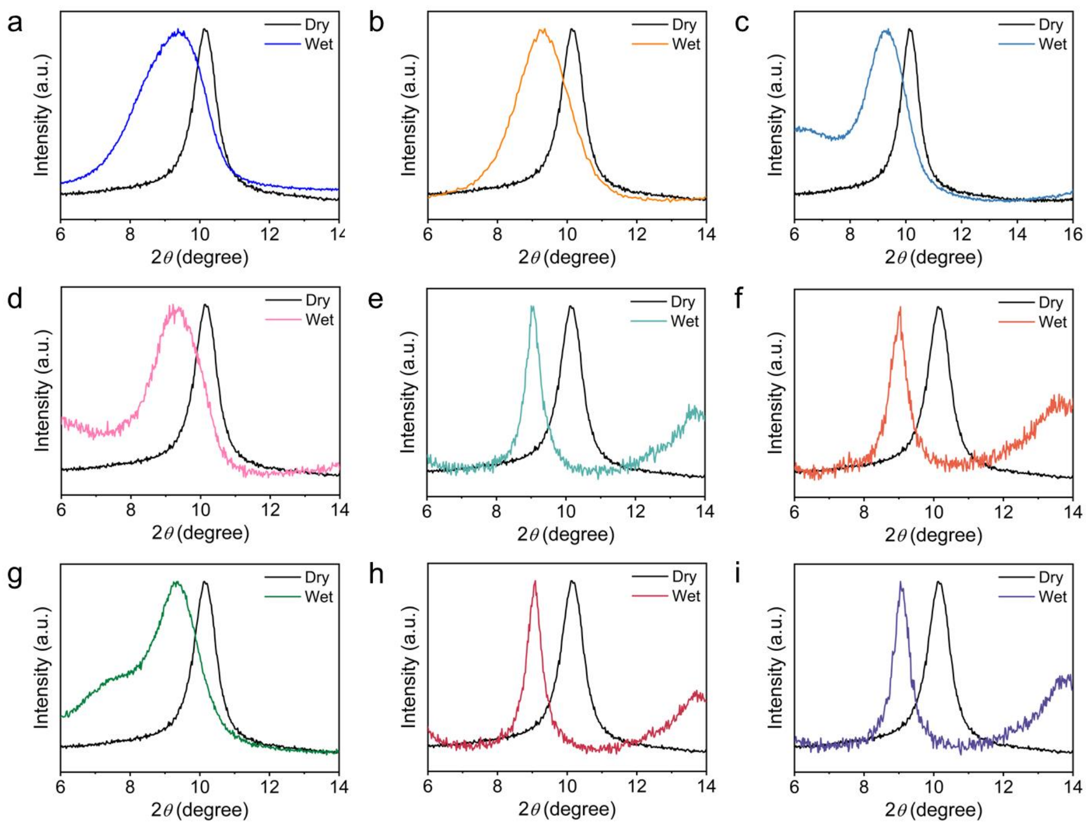

Figure S3. XRD patterns of GO membranes in dry and wet state. Compared to the dry-state d-spacing, the wet-state d-spacing swelling in ethanol (**a**), propanol (**b**), butanol (**c**), 3-methyl-1-butanol (**d**), 1-pentanol (**e**), 1-hexyl alcohol (**f**), isobutyl alcohol (**g**), 2-methyl-1-pentanol (**h**), and 4-methyl-1-pentanol (**i**) expanded.

a

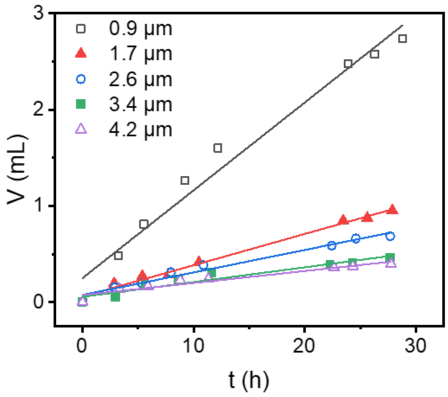

b

Thickness of GO membranes	The slope of the reactant flow volume as a function of time, k
0.9 μm	0.091 $mL \cdot h^{-1}$
1.7 μm	0.033 $mL \cdot h^{-1}$
2.6 μm	0.023 $mL \cdot h^{-1}$
3.4 μm	0.015 $mL \cdot h^{-1}$
4.2 μm	0.012 $mL \cdot h^{-1}$

Figure S4. a) Flow curves of reaction solution of styrene oxide and isopropyl alcohol corresponding to GO membranes with different thickness. Flow curves of reaction solution is nearly linear and reaction solution flow slows down nonlinearly with increasing GO membranes thickness. **b**) For GO membranes with different thicknesses, the corresponding slope of the reactant flow volume as a function of time, k.

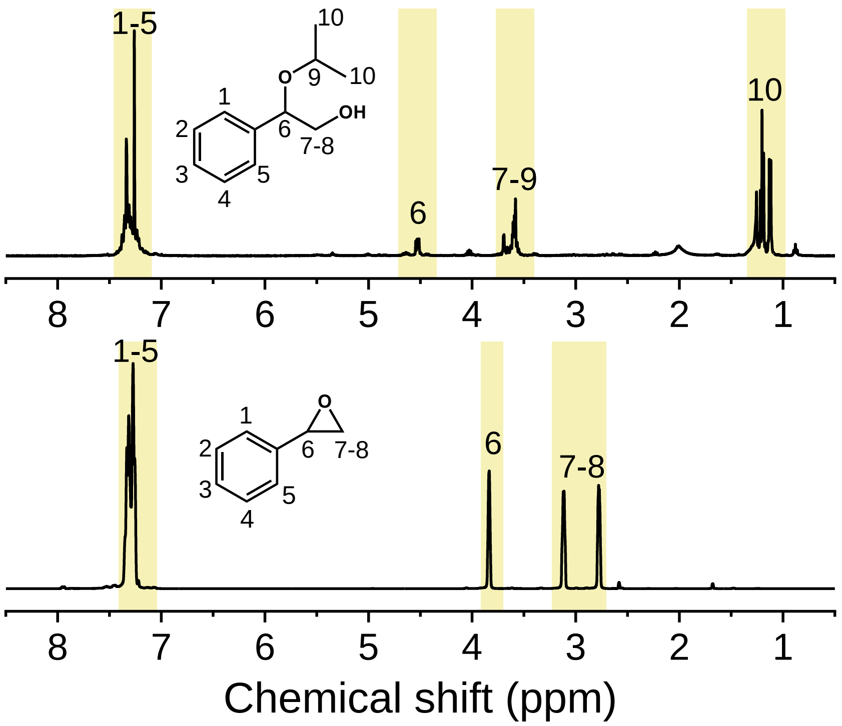

Figure S5. ^{1}H NMR spectra and characteristic protons of 2-isopropoxy-2-phenylethan-1-ol (product) and styrene oxide (reactant).

2-isopropoxy-2-phenylethan-1-ol. ^{1}H NMR (600 MHz, $CDCl_3$) δ 7.43 – 7.27 (m, 5H), 4.53 (dd, J = 7.9, 4.7 Hz, 1H), 3.65 – 3.52 (m, 3H), 1.24 – 1.06 (m, 6H).

Styrene oxide. ^{1}H NMR (600 MHz, $CDCl_3$) δ 7.30 (dt, J = 24.0, 7.6 Hz, 5H), 3.84 (d, J = 4.3 Hz, 1H), 3.12 (d, J = 4.5 Hz, 1H), 2.81 – 2.74 (m, 1H).

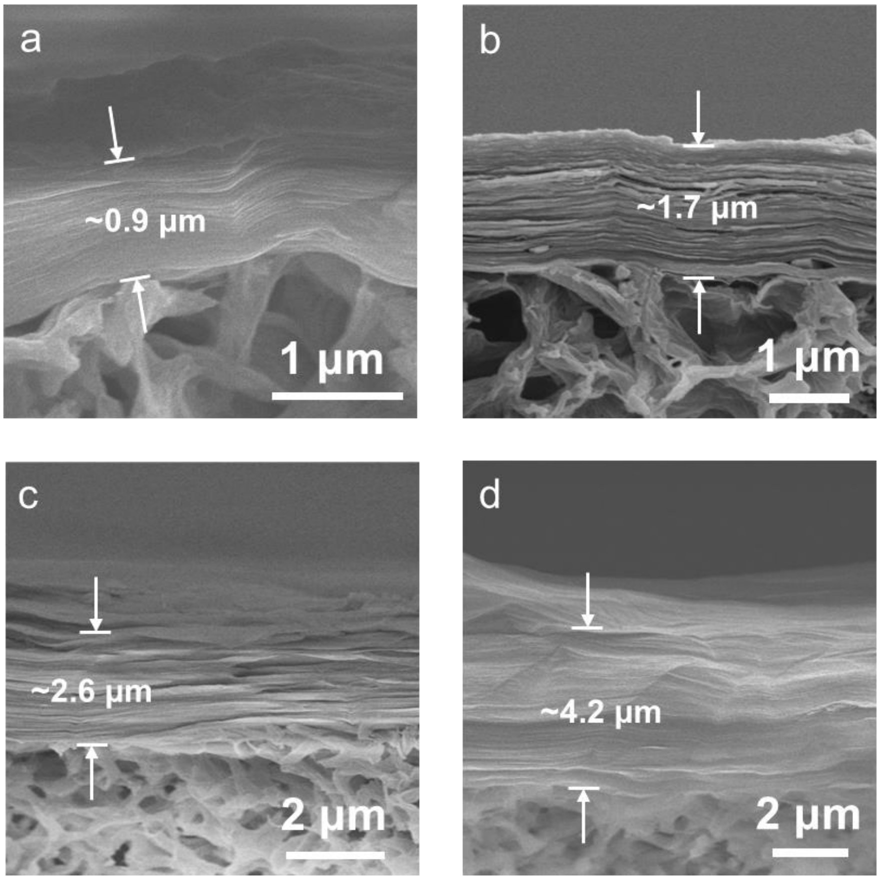

Figure S6. SEM observation of cross-sections of the GO membrane with different thicknesses, showing compact multilayer structures.

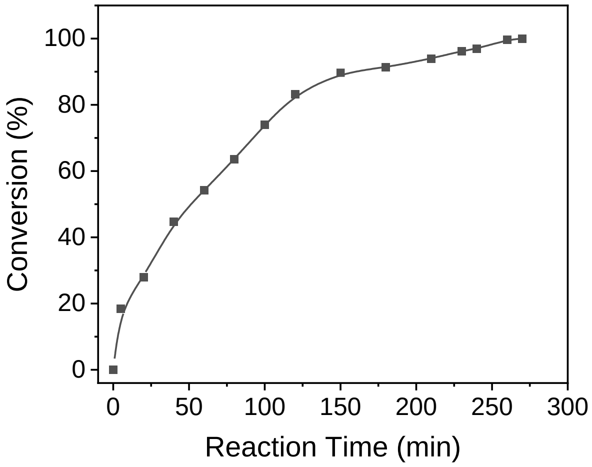

Figure S7. Conversion of bulk reaction of styrene oxide with i-propanol catalyzed by GO nanosheets corresponding to time. The conversion of the bulk reaction increases with reaction time, and nearly complete conversion is achieved at ~270 min (~4.5 h).

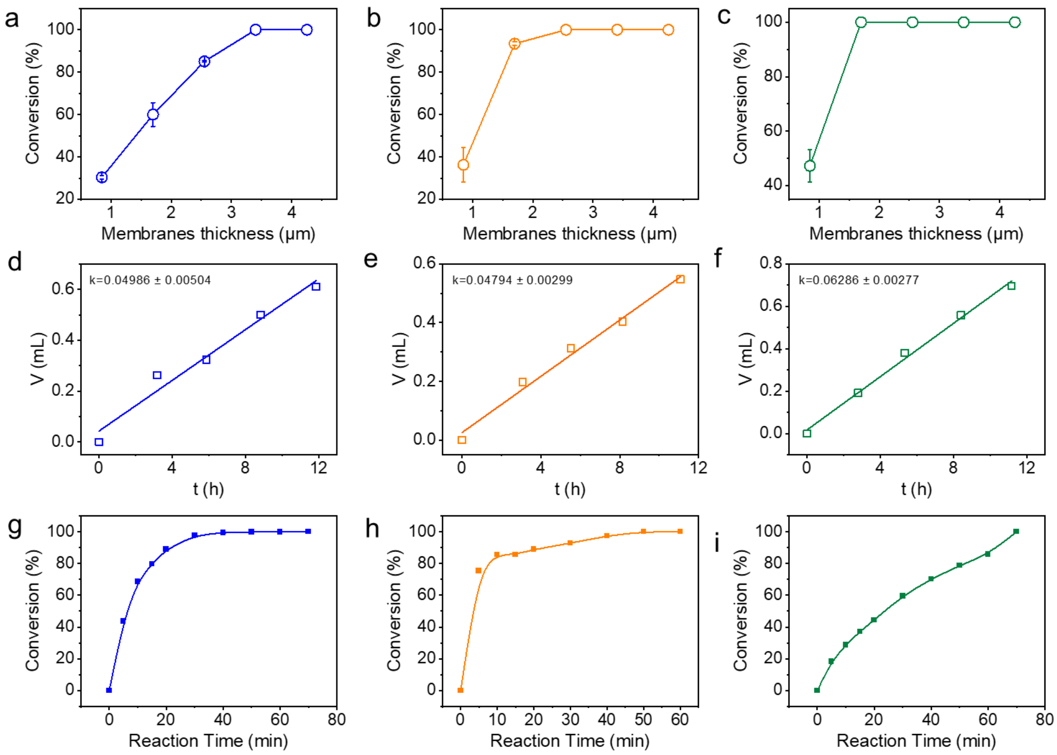

Figure S8. Comparison of confined reactions and bulk reactions. (**a-c**) Conversion of confined ring-opening reactions between styrene oxide and ethanol (**a**), propanol (**b**), and isobutyl alcohol (**c**) as a function of GO membranes thickness. Nearly 100% conversion was achieved at the membrane thickness of 3.4 µm, 2.6 µm, and 1.7 µm, respectively. (**d-f**) Flow curves of reaction solution of styrene oxide and ethanol (**d**), propanol (**e**), and isobutyl alcohol (**f**) through GO membranes with the thickness of 3.4 µm, 2.6 µm, and 1.7 µm, respectively. (**g-i**) Conversion of bulk ring-opening reactions of styrene oxide with ethanol (**g**), propanol (**h**), and isobutyl alcohol (**i**) catalyzed by GO nanosheets corresponding to time.

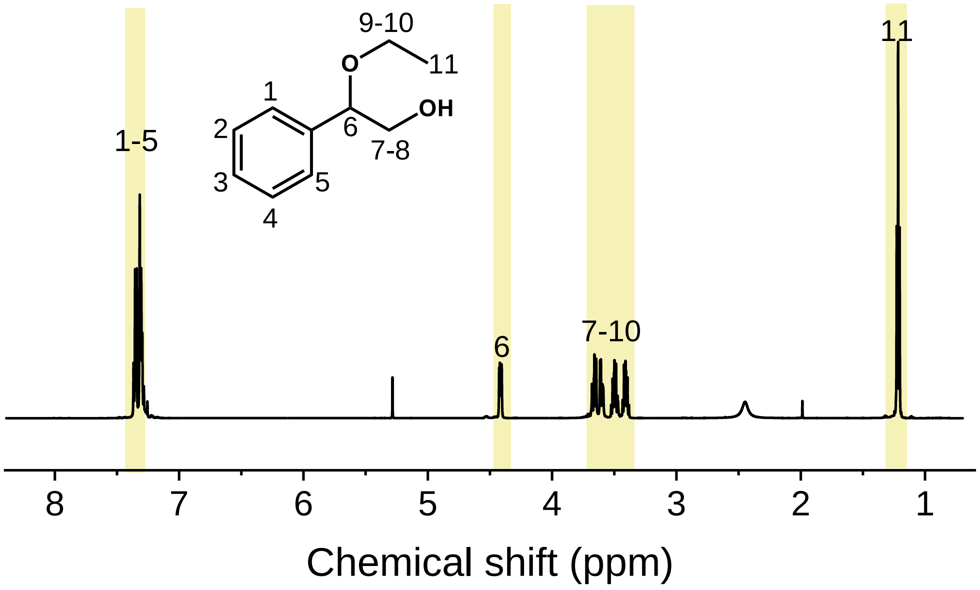

Figure S9. The ^{1}H NMR spectra of the permeation mixture obtained from the reaction solution of styrene oxide and ethanol through GO membranes with a thickness of 3.4 μm.

2-ethoxy-2-phenylethan-1-ol. ^{1}H NMR (600 MHz, $CDCl_3$): δ 7.38 – 7.25 (m, 5H), 4.42 (dd, J = 8.6, 3.7 Hz, 1H), 3.74 – 3.55 (m, 2H), 3.55 – 3.35 (m, 2H), 1.22 (t, J = 7.0 Hz, 3H).

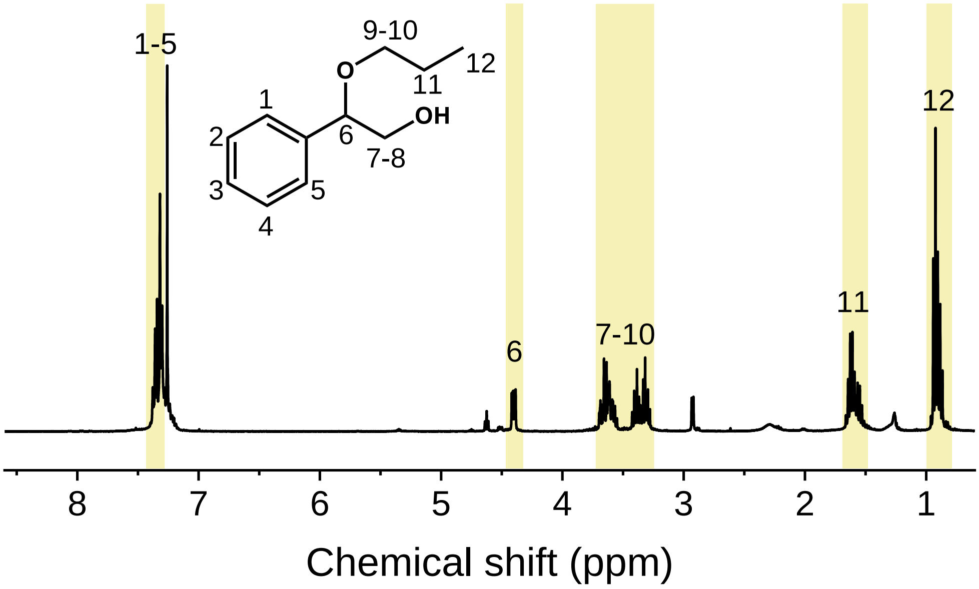

Figure S10. The ^{1}H NMR spectra of the permeation mixture obtained from the reaction solution of styrene oxide and propanol through GO membrane with a thickness of 2.6 μm.

2-phenyl-2-propoxyethan-1-ol. ^{1}H NMR (600 MHz, $CDCl_3$) δ 7.39 – 7.28 (m, 5H), 4.40 (dd, J = 8.7, 3.8 Hz, 1H), 3.72 – 3.54 (m, 2H), 3.45 – 3.26 (m, 2H), 1.62 (dd, J = 14.1, 7.4, 6.6 Hz, 2H), 0.93 (dt, J = 11.1, 7.4 Hz, 3H).

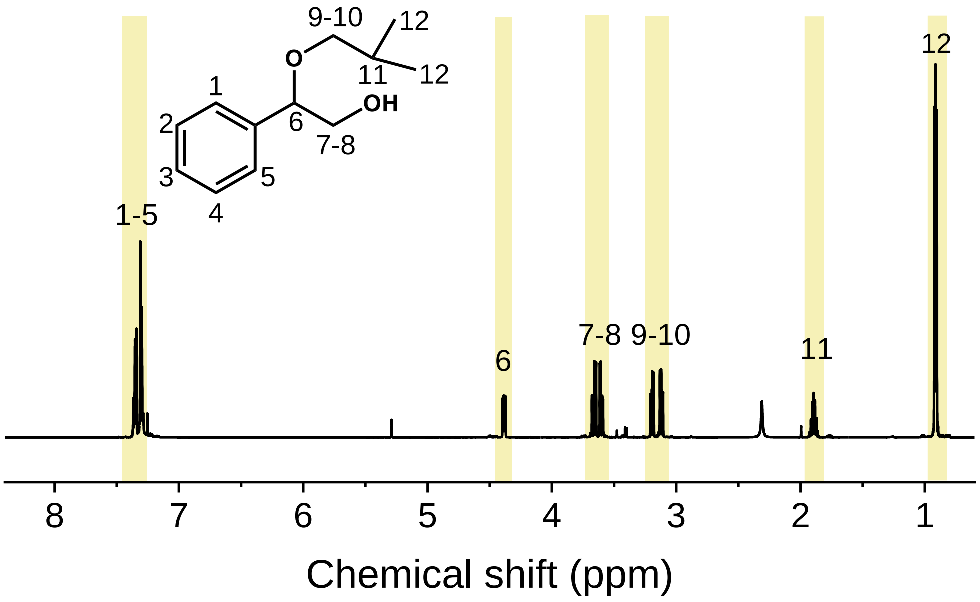

Figure S11. The ^{1}H NMR spectra of the permeation mixture obtained from the reaction solution of styrene oxide and isobutyl alcohol through GO membrane with a thickness of 1.7 μm.

2-isobutoxy-2-phenylethan-1-ol. ^{1}H NMR (600 MHz, $CDCl_3$) δ 7.43 – 7.18 (m, 5H), 4.38 (dd, 1H), 3.66 - 3.60 (dd, 2H), 3.19 -3.12 (dd, 2H), 1.89 (dh, J = 13.4, 6.7 Hz, 1H), 0.91 (dd, J = 6.7, 4.5 Hz, 6H)

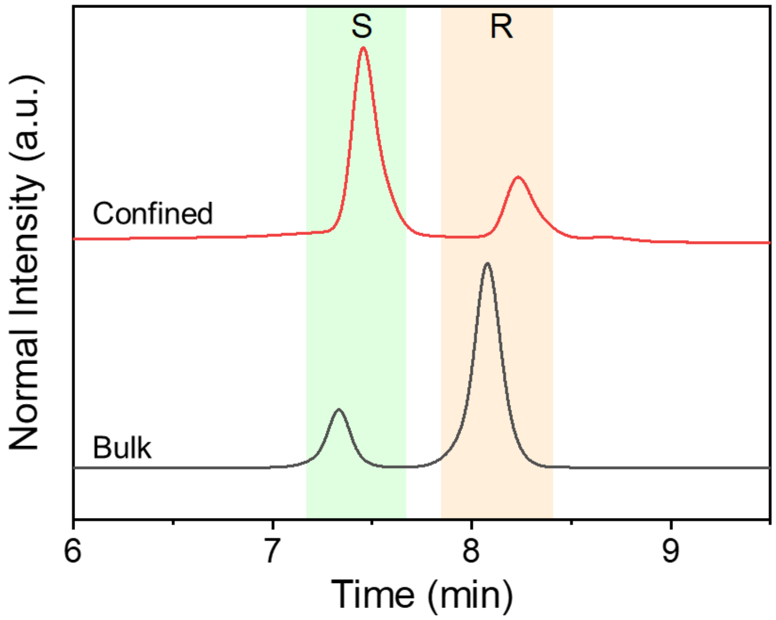

Figure S12. The HPLC traces of the permeation mixture obtained from the commercial (S)-styrene oxide and isopropanol under confined reactions (top) or bulk reactions (bottom). (acetonitrile/water 4:6; flow rate 0.5 mL·min^{-1}; λ = 205 nm; $t_{(S)}$ = 7.5 min; $t_{(R)}$ =8.3 min).

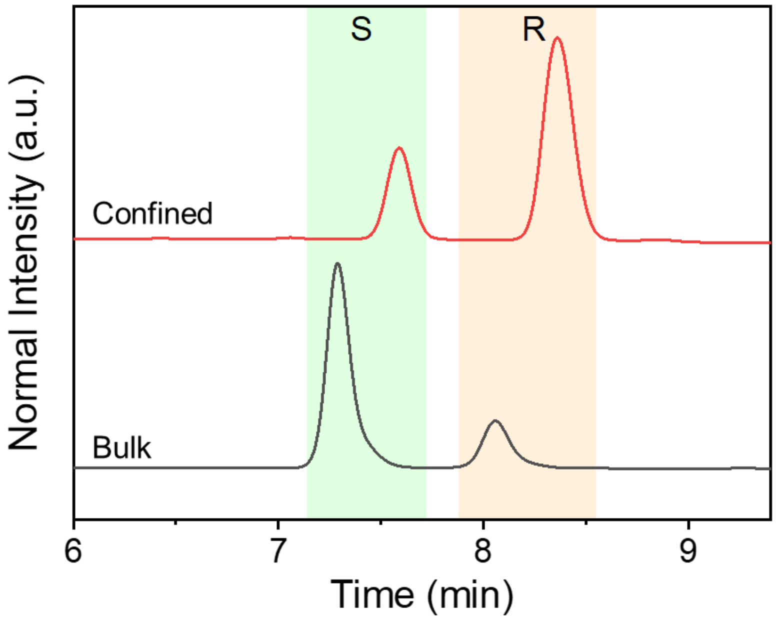

Figure S13. The HPLC traces of the permeation mixture obtained from the commercial (R)-styrene oxide and isopropanol under confined reactions (top) or bulk reactions (bottom). As for confined reactions, the S/R enantiomeric ratio was determined to be 26:74 by chiral HPLC analysis while S/R enantiomeric ratio was determined to be 80:20 under bulk reactions (acetonitrile/water 4:6; flow rate 0.5 mL·min^{-1}; λ = 205 nm; $t_{(S)}$ = 7.5 min; $t_{(R)}$ = 8.3 min).

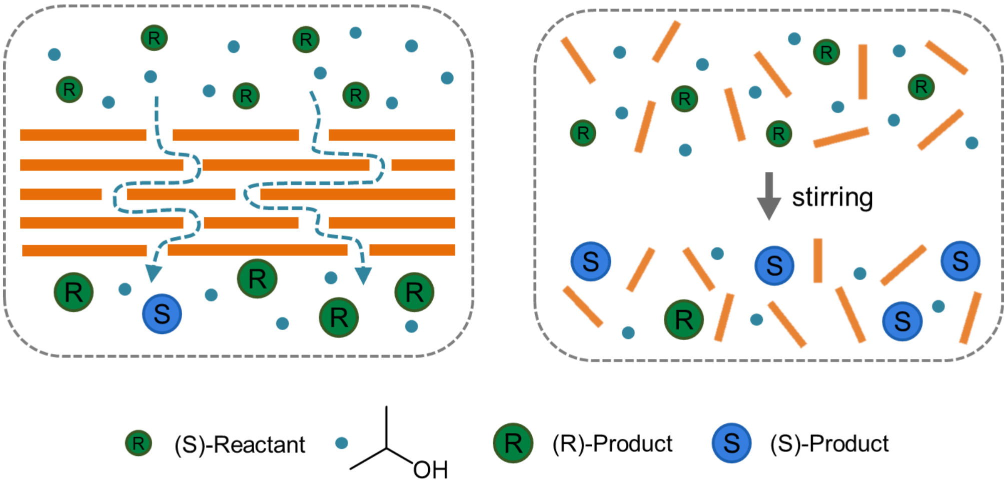

Figure S14. Schematic illustration showing the difference about stereoselective reaction of (R)-styrene oxide between confined reactions and bulk reactions.

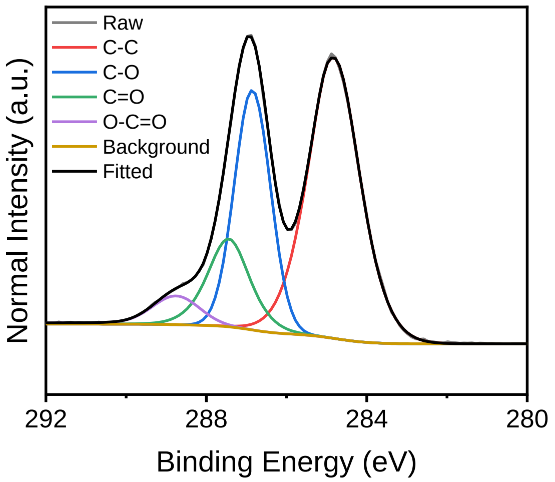

Figure S15. C 1s XPS spectra of the GO-120 °C. The peaks at approximately 284.8, 286.8, 287.4 and 288.7 eV were attributed to the C–C, C–O, C=O (carbonyl C) and O=C–OH (carboxy C) functional groups, respectively.

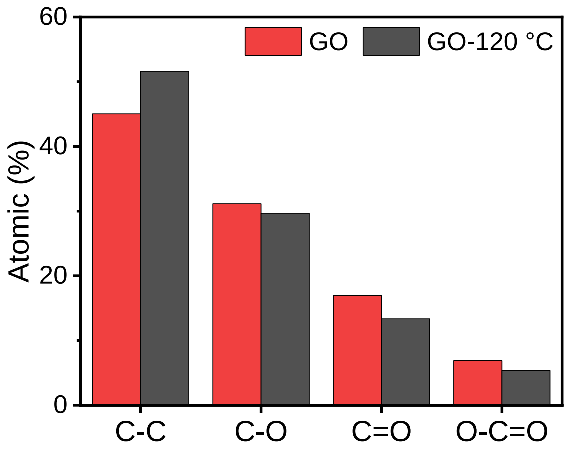

Figure S16. The relative ratio of various carbon atoms in the high-resolution C 1s spectra of GO and GO-120 °C membranes. Compared to GO membranes, GO-120 °C membranes showed an increase in C-C groups atomic ratio and a decrease in C-O, C=O and O-C=O groups atomic ratio.

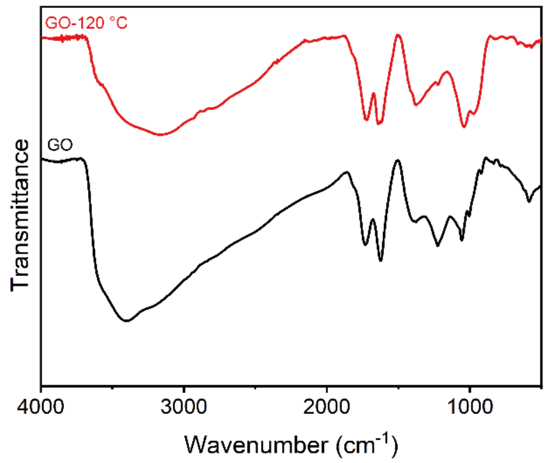

Figure S17. FTIR spectra of GO-120 °C membrane compared to GO membrane. the peak at around 3408 cm^{-1} attributed to the -OH stretching vibration of GO-120 °C membrane is weaker than that of the GO membranes, indicating the partly disappearance of hydroxyl groups.

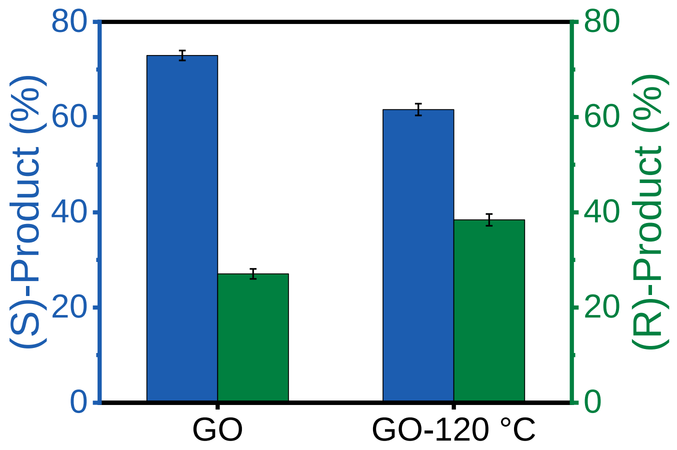

Figure S18. Percentage of (R)-product and (S)-product obtained under confined reactions, catalyzed by GO and GO-120 °C membranes.

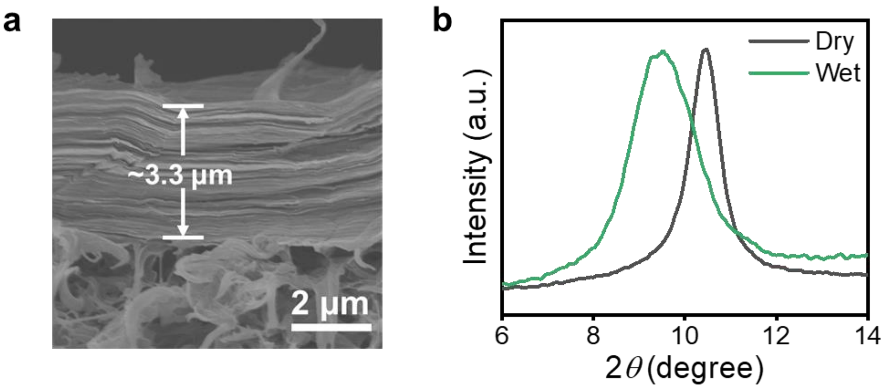

c

Sample	Wet state 2θ (degree)	Wet state d (Å)
GO	9.23	9.57
GO-120 °C	9.48	9.32

Figure S19. a) SEM observation of cross-section of the GO-120 °C membrane. b) XRD Characterization of GO-120 °C membrane in dry and wet state. c)The 2θ degree and the corresponding wet-state interlayer diffraction spacing of GO and GO-120 °C membranes swelling in isopropanol.

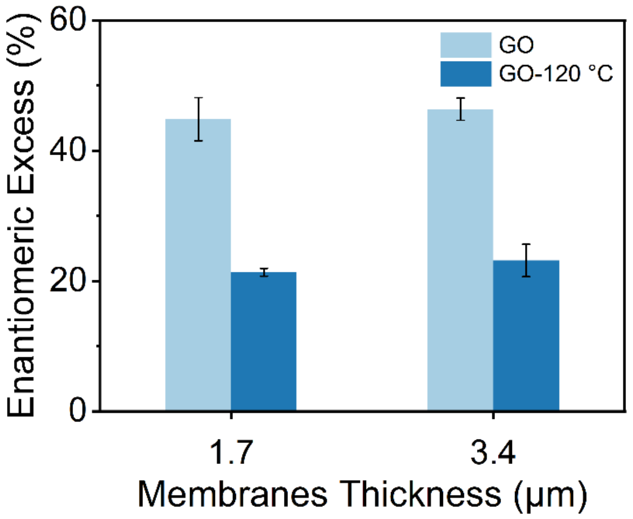

Figure S20. Enantiomeric excess of ring-opening reactions between (S)-styrene oxide and isopropanol catalyzed by GO membranes and GO-120 °C membranes with thicknesses of 1.7 μm and 3.4 μm.

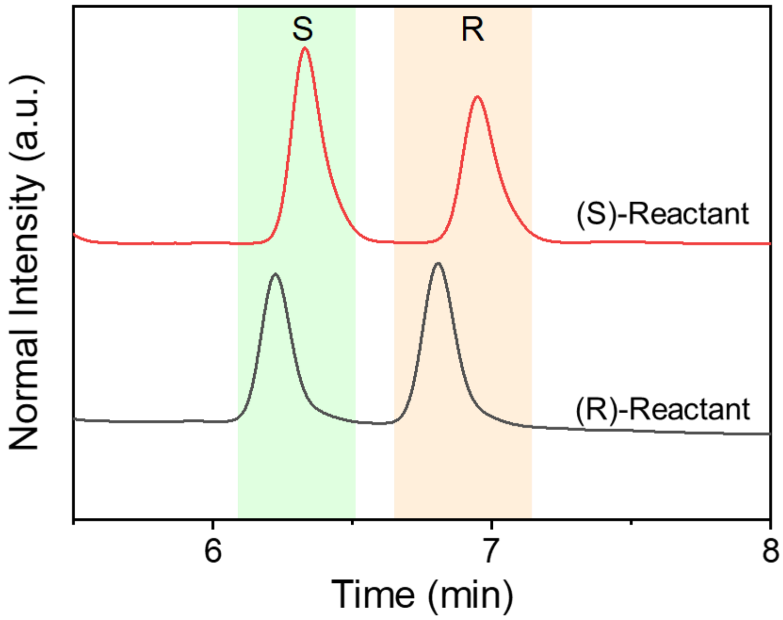

Figure S21. The HPLC traces of the permeation mixture of (S)-styrene oxide (top) or (R)-styrene oxide (bottom) reacting with ethanol, respectively, under confined reactions. As for (S)-styrene oxide, the S/R enantiomeric ratio was determined to be 55:45 by chiral HPLC analysis (acetonitrile/water 4:6; flow rate 0.5 mL·min^{-1}; λ = 205 nm; $t_{(S)}$ = 6.3 min; $t_{(R)}$ = 6.9 min).

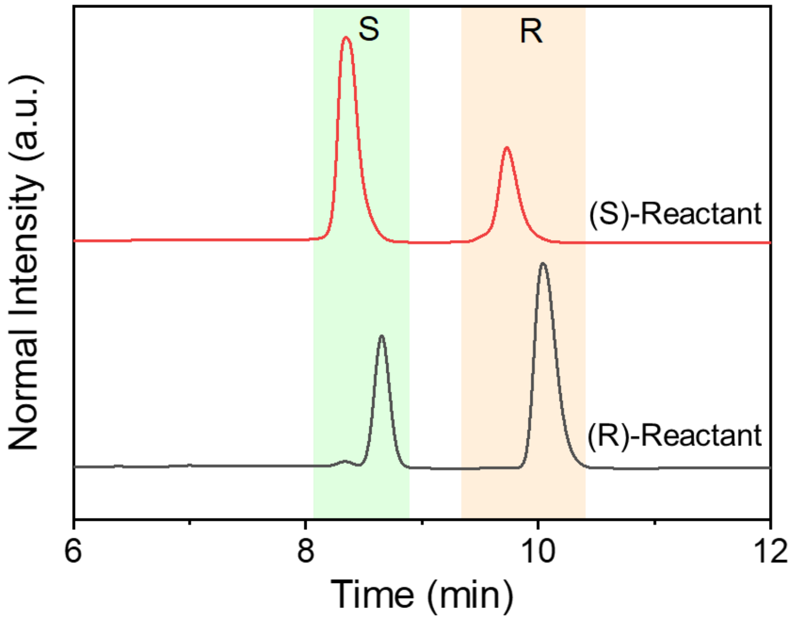

Figure S22. The HPLC traces of the permeation mixture of (S)-styrene oxide (top) or (R)-styrene oxide (bottom) reacting with propanol, respectively, under confined reactions. As for (S)-styrene oxide, the S/R enantiomeric ratio was determined to be 68:32 by chiral HPLC analysis (acetonitrile/water 4:6; flow rate 0.5 mL·min^{-1}; λ = 205 nm; $t_{(S)}$ = 8.4 min; $t_{(R)}$ = 9.6 min).

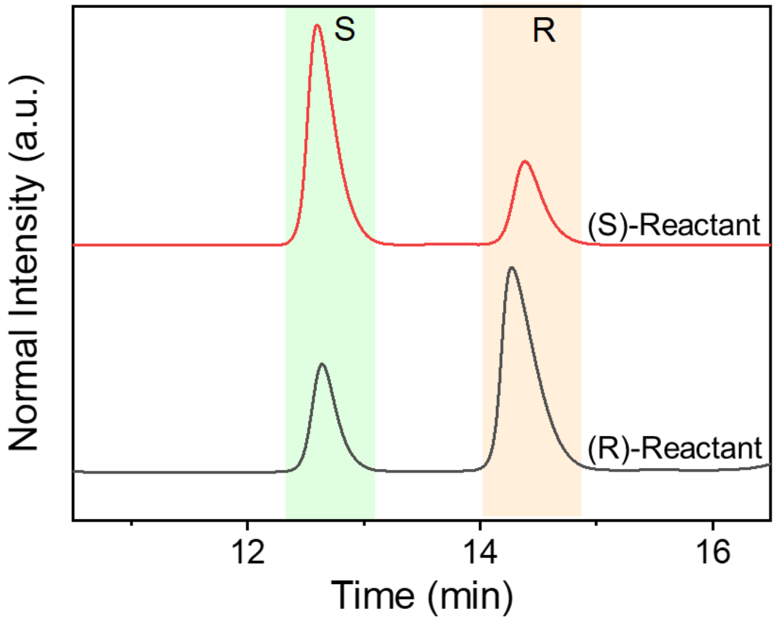

Figure S23. The HPLC traces of the permeation mixture of (S)-styrene oxide (top) or (R)-styrene oxide (bottom) reacting with 1-butanol, respectively, under confined reactions. As for (S)-styrene oxide, the S/R enantiomeric ratio was determined to be 71:29 by chiral HPLC analysis (acetonitrile/water 4:6; flow rate 0.5 mL·min^{-1}; λ = 205 nm; $t_{(S)}$ = 12.6 min; $t_{(R)}$ = 14.4 min).

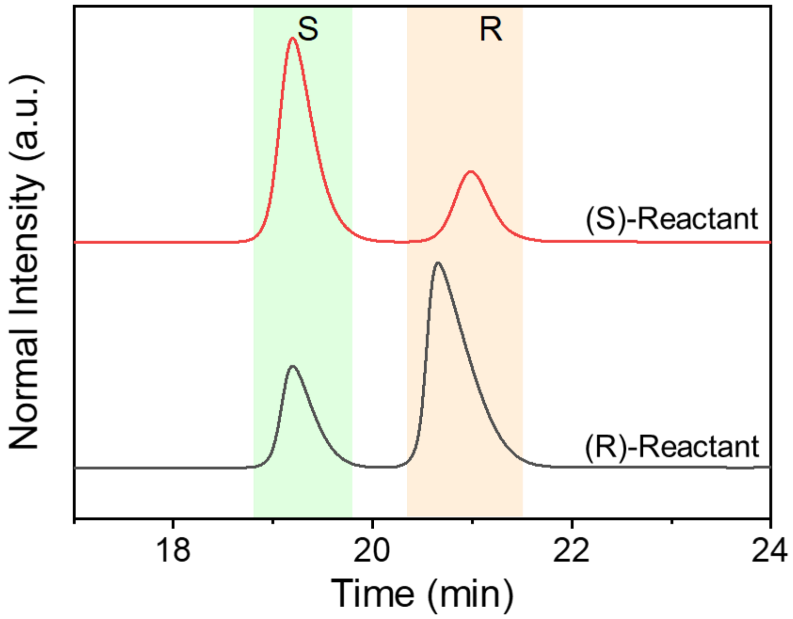

Figure S24. The HPLC traces of the permeation mixture obtained from the commercial (S)-styrene oxide and 3-methyl-1-butanol under confined reactions. As for (S)-styrene oxide, the S/R enantiomeric ratio was determined to be 73:27 by chiral HPLC analysis (acetonitrile/water 4:6; flow rate 0.5 mL·min^{-1}; λ = 205 nm; $t_{(S)}$ = 19.2 min; $t_{(R)}$ = 21.0 min).

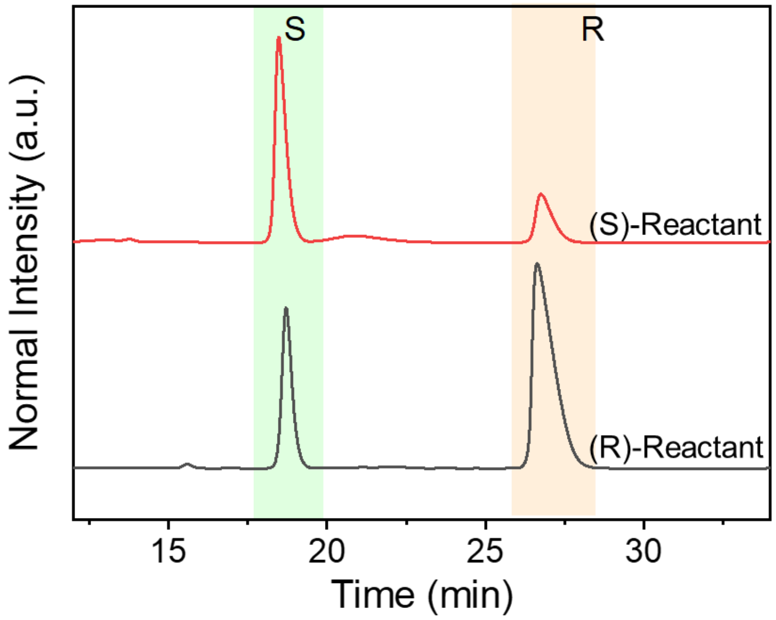

Figure S25. The HPLC traces of the permeation mixture of (S)-styrene oxide (top) or (R)-styrene oxide (bottom) reacting with 1-pentanol, respectively, under confined reactions. As for (S)-styrene oxide, the S/R enantiomeric ratio was determined to be 73:27 by chiral HPLC analysis (acetonitrile/water 4:6; flow rate 0.5 mL·min^{-1}; λ = 205 nm; $t_{(S)}$ = 18.5 min; $t_{(R)}$ = 26.8 min).

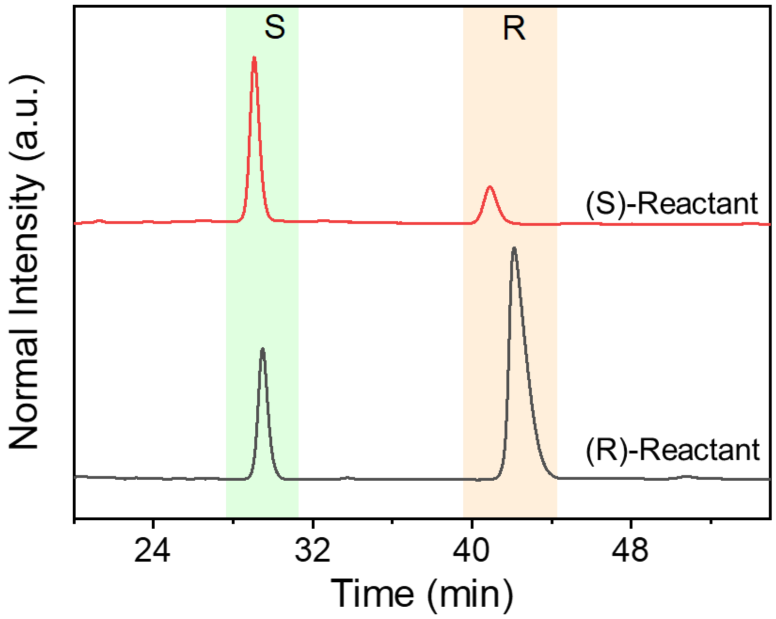

Figure S26. The HPLC traces of the permeation mixture of (S)-styrene oxide (top) or (R)-styrene oxide (bottom) reacting with 1-hexanol, respectively, under confined reactions. As for (S)-styrene oxide, the S/R enantiomeric ratio was determined to be 75:25 by chiral HPLC analysis (acetonitrile/water 4:6; flow rate 0.5 mL·min^{-1}; λ = 205 nm; $t_{(S)}$ = 27.1 min; $t_{(R)}$ = 38.9 min).

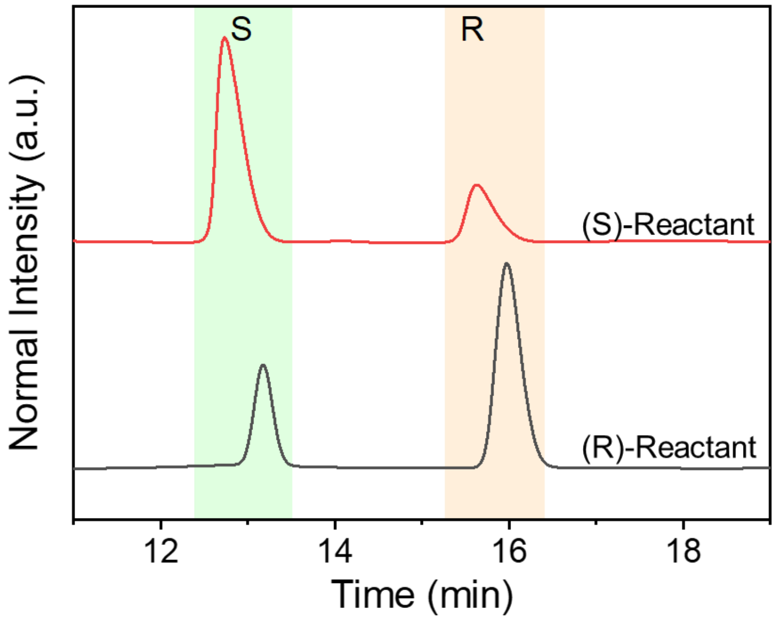

Figure S27. The HPLC traces of the permeation mixture of (S)-styrene oxide (top) or (R)-styrene oxide (bottom) reacting with isobutyl alcohol, respectively, under confined reactions. As for (S)-styrene oxide, the S/R enantiomeric ratio was determined to be 76:24 by chiral HPLC analysis (acetonitrile/water 4:6; flow rate 0.5 mL·min^{-1}; λ = 205 nm; $t_{(S)}$ = 12.7 min; $t_{(R)}$ = 15.6 min).

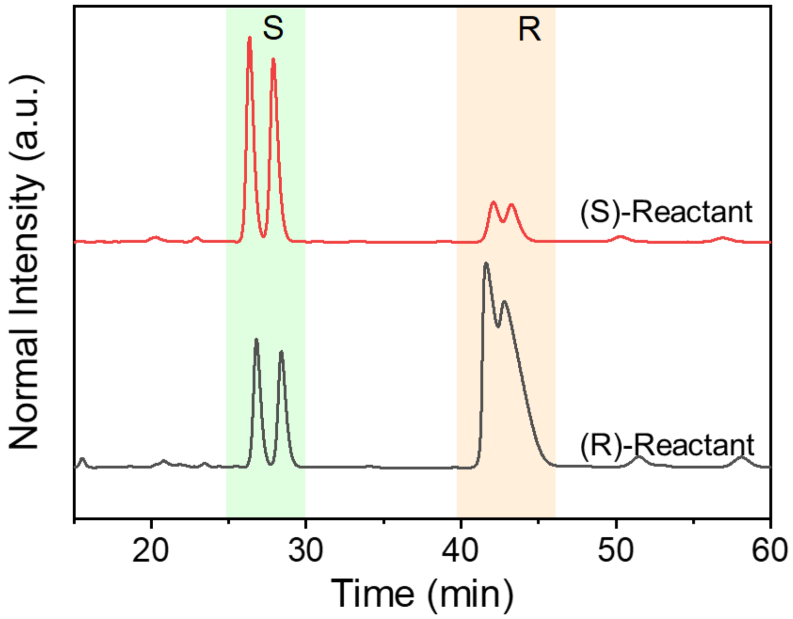

Figure S28. The HPLC traces of the permeation mixture obtained from the commercial (S)-styrene oxide (top) or (R)-styrene oxide (bottom) reacting with 2-methyl-1-pentanol, respectively, under confined reactions. As for (S)-styrene oxide, the S/R enantiomeric ratio was determined to be 80:20 by chiral HPLC analysis (acetonitrile/water 4:6; flow rate 0.5 mL·min^{-1}; λ = 205 nm; $t_{(S)}$ = 27.0 min; $t_{(R)}$ = 43.2 min).

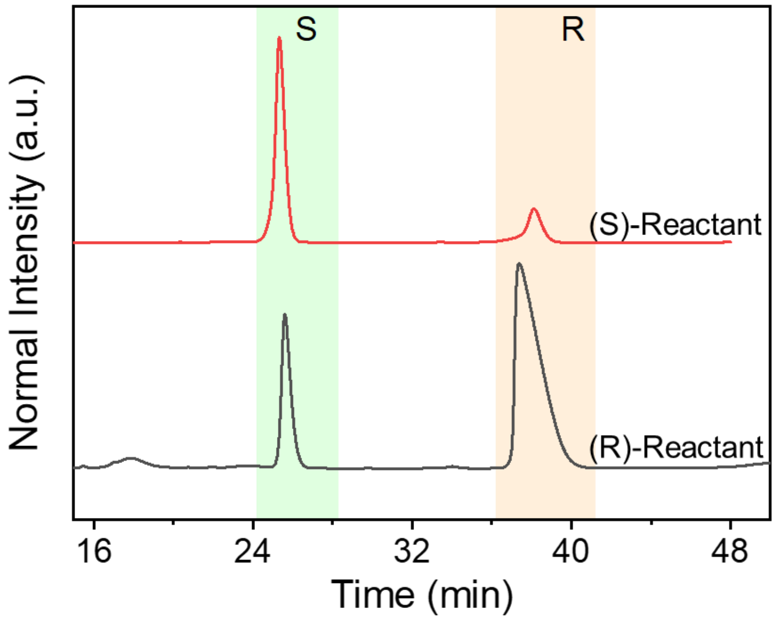

Figure S29. The HPLC traces of the permeation mixture obtained from the commercial (S)-styrene oxide (top) or (R)-styrene oxide (bottom) reacting with 4-methyl-1-pentanol, respectively, under confined reactions. As for (S)-styrene oxide, the S/R enantiomeric ratio was determined to be 80:20 by chiral HPLC analysis (acetonitrile/water 4:6; flow rate 0.5 mL·min^{-1}; λ = 205 nm; $t_{(S)}$ = 25.6 min; $t_{(R)}$ = 38.4 min).

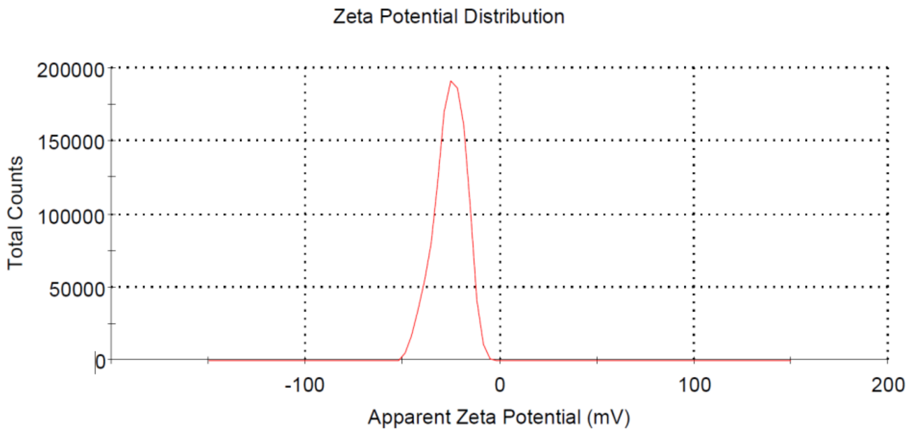

Figure S30. The zeta potential of GO suspension (0.20 mg·mL^{-1}) is -25.6 mV, which proved the negatively charged environment on the GO surface.

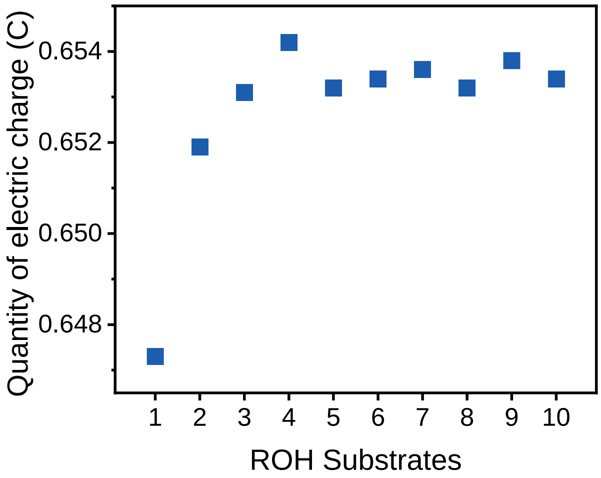

Figure S31. DFT calculations of electric charge of different alkyl-oxygen (RO) groups.

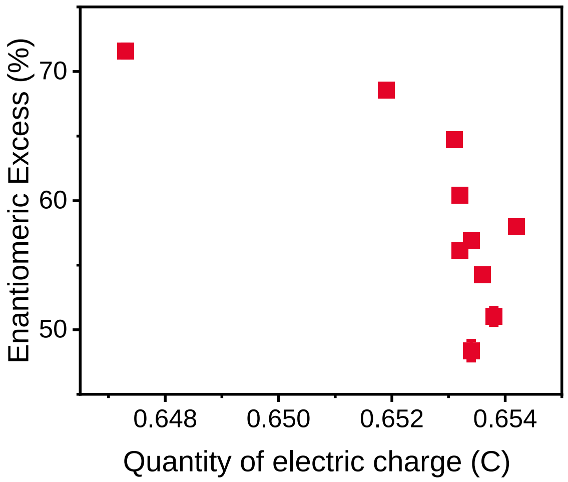

Figure S32. Enantiomeric excess of ring-opening reactions between S-styrene oxide and alkyl alcohols under bulk conditions as a function of electric charges of different RO substituents.

Table S1. The 2θ degree and the corresponding wet-state d-spacing swelling in ethanol, propanol, butanol, 3-methyl-1-butanol, 1-pentanol, 1-hexyl alcohol, isobutyl alcohol, 2-methyl-1-pentanol, and 4-methyl-1-pentanol of GO membranes.

Solvent	Wet state<br>2θ (degree)	Wet state<br>d (Å)
**Ethanol**	9.28	9.52
**Propanol**	9.30	9.50
**Butanol**	9.02	9.80
**3-Methyl-1-Butanol**	9.32	9.49
**1-Pentanol**	9.05	9.76
**1-Hexyl Alcohol**	9.02	9.79
**Isobutyl Alcohol**	9.34	9.46
**2-Methyl-1-Pentanol**	9.07	9.75
**4-Methyl-1-Pentanol**	9.09	9.72

Table S2. The relative atomic ratio of various carbon atoms in the high-resolution C 1*s* spectra of GO and GO-120 °C membranes.

**Sample**	**C-C 284.8 eV**	**C-O 286.8 eV**	**C=O (carbonyl C) 287.4 eV**	**O=C–OH (carboxy C) 288.7 eV**
**GO**	45.04	31.13	16.93	6.89
**GO-120 °C**	51.62	29.67	13.35	5.36

Supplemental References

[1] X. Li, Q. Chen, X. Tong, S. Zhang, H. Liu, *J. Membr. Sci.* **2021**, *634*, 119350.

[2] C. Meng, Y. Sheng, Q. Chen, H. Tan, H. Liu, *J. Membr. Sci.* **2017**, *526*, 25.

[3] H.-R. Chae, J. Lee, C.-H. Lee, I.-C. Kim, P.-K. Park, *J. Membr. Sci.* **2015**, *483*, 128.

[4] O. C. Compton, D. A. Dikin, K. W. Putz, L. C. Brinson, S. T. Nguyen, *Adv. Mater.* **2010**, *22*, 892.

[5] J. Ji, Q. Kang, Y. Zhou, Y. Feng, X. Chen, J. Yuan, W. Guo, Y. Wei, L. Jiang, *Adv. Funct. Mater.* **2017**, *27*, 1603623.

[6] R. L. G. Lecaros, K. M. Deseo, W.-S. Hung, L. L. Tayo, C.-C. Hu, Q.-F. An, H.-A. Tsai, K.-R. Lee, J.-Y. Lai, *J. Membr. Sci.* **2019**, *576*, 36.
